# Adolescents’ heightened risk-seeking in a probabilistic gambling task

**DOI:** 10.1016/j.cogdev.2009.11.003

**Published:** 2010-04

**Authors:** Stephanie Burnett, Nadège Bault, Giorgio Coricelli, Sarah-Jayne Blakemore

**Affiliations:** a UCL Institute of Cognitive Neuroscience, 17 Queen Square, London WC1N 3AR, UK; b UCL Institute of Neurology, Queen Square, London WC1N 3BG, UK; c Cognitive Neuroscience Centre, CNRS UMR 5229, 67 Boulevard Pinel, Bron 69675 Cedex, France

**Keywords:** Adolescence, Development, Decision-making, Risk, Emotion

## Abstract

This study investigated adolescent males’ decision-making under risk, and the emotional response to decision outcomes, using a probabilistic gambling task designed to evoke counterfactually mediated emotions (relief and regret). Participants were 20 adolescents (aged 9–11), 26 young adolescents (aged 12–15), 20 mid-adolescents (aged 15–18) and 17 adults (aged 25–35). All were male. The ability to maximize expected value improved with age. However, there was an inverted U-shaped developmental pattern for risk-seeking. The age at which risk-taking was highest was 14.38 years. Although emotion ratings overall did not differ across age, there was an increase between childhood and young adolescence in the strength of counterfactually mediated emotions (relief and regret) reported after receiving feedback about the gamble outcome. We suggest that continuing development of the emotional response to outcomes may be a factor contributing to adolescents’ risky behaviour.

Adolescents tend to engage in ‘risky’ behaviours—those with high subjective desirability but high potential for harm (Geier & Luna, 2009). These include dangerous driving, unsafe sex and experimentation with addictive substances (Eaton et al., 2008). This phenomenon has led to the suggestion that adolescents are risk-seeking in general (Casey, Jones, & Hare, 2008; Steinberg, 2008), where risk-seeking can be conceptualised as a behavioural economic preference for decisions with unsure outcomes (Knight, 1921). However, engagement in risky behaviours is likely to have a variety of social, emotional and cognitive causes, and few empirical studies have directly compared risk-seeking in children, adolescents and adults using laboratory-based behavioural tasks (Crone, Bullens, van der Plas, Kijkuit, & Zelazo, 2008; Harbaugh, Krause, & Vesterlund, 2002; Van Leijenhorst, Westeberg, & Crone, 2008; see Boyer, 2006, for review). In the current study, we investigated the development of risk-seeking between late childhood (age 9) and adulthood (age 35), using a behavioural economic task that involved making gambling decisions in an emotional context. We expected to observe heightened risk-seeking in adolescents relative to adults, in line with previous empirical data (Eshel, Nelson, Blair, Pine, & Ernst, 2007; Figner, Mackinlay, Wilkening, & Weber, 2009). In line with theoretical suggestions (Casey et al., 2008; Steinberg, 2008), as well as recent empirical findings (O’Brien & Steinberg, 2009), we hypothesised that adolescents would also show heightened risk-seeking relative to children.

We also examined age differences in emotional responses to gamble outcomes. Choices are influenced by expected gains—the economist's “expected value” (EV), i.e., the summed values of potential outcomes weighted by their respective probabilities. However, decisions are also influenced by how one expects to *feel* afterwards. As an example, consider the phenomenon of postcode lotteries. Players pay a small amount of money each week to buy the chance of winning a large sum of money if their postcode is drawn from a pool containing all the postcodes in the country. The EV of playing is negative (i.e., you can expect to lose money if you play), since the chance of winning is very small. However, a great many people play, and it is thought that their doing so is partly due to emotion. People anticipate that they will feel very bad if their postcode is drawn and they did not buy a ticket. They will feel *regret* (as well as envy, if a neighbor sharing their postcode bought a ticket). The emotion of regret is a counterfactually mediated emotion, as it arises due to counterfactual comparison between what is and the more desirable outcome that might have been if a different decision had been made. The positive counterpart of regret is relief (when the counterfactual comparison favors what is). Anticipation of counterfactually mediated emotions such as relief and regret may influence decision-making. Studies involving gambling tasks have shown deviations from pure EV-maximising behaviour attributable to participants taking into account the regret their decisions may cause them to feel. They are prepared to accept smaller rewards to avoid putting themselves at risk of experiencing the unpleasant emotion of regret (Camille et al., 2004; Coricelli et al., 2005; Coricelli, Dolan, & Sirigu, 2007).

Adolescents are thought to show exaggerated or more labile emotional responses to outcomes compared to children or adults (Casey et al., 2008; Ernst & Mueller, 2007; Ernst et al., 2005; Eshel et al., 2007). Also, the ability to think counterfactually about the outcomes of decisions may continue to develop during adolescence (Baird & Fugelsang, 2004). If the anticipation of counterfactually mediated emotions plays a key role in decision-making in adults (Coricelli et al., 2007), immaturities in the genesis or use of counterfactually mediated emotions such as relief and regret during decision-making could contribute to risky decision-making in adolescents (Steinberg, 2008). To our knowledge, no previous study has directly compared the risk-seeking, and associated emotions of relief and regret, of children and adults.

In the present study, participants aged 9–35 years engaged in a probabilistic gambling task (Camille et al., 2004; Coricelli et al., 2005) in which paired gambles differed in EV, in risk, and in the potential to generate counterfactually mediated emotions (relief and regret). In this type of task, an optimal decision-maker should choose gambles with greater EV, to maximise winnings. However, gambles with the same EV can differ in their level of risk, where risk is measured as the outcome variance of a gamble (Bossaerts & Hsu, 2008). We used outcome variance and EV of gambles to identify participants’ utility function (Bossaerts & Hsu, 2008).

Our first goal in the current study was to investigate the impact of EV on choice across age. Although EV maximisation may continue to develop, we predicted that even the youngest participants would tend to choose gambles with higher EV, since it has been shown that children as young as age 5 show a rudimentary sensitivity to EV (Schlottmann, 2001). When 5-year-olds view probabilistic events associated with different prizes won by a puppet, the level of happiness children judge the puppet will achieve is proportional to the value of a prize multiplied by its approximate probability, that is, the approximate EV. However, although probability and value judgment may be mature by mid-adolescence (Boyer, 2006), the use of such information to guide behaviour in emotional contexts, as in the current paradigm, may still be developing. In a behavioural economic study by Harbaugh et al. (2002), in which participants aged 5–64 years made a series of choices between a certain outcome and a gamble with equivalent EV, the proportion of unbiased, objective decisions increased with age. Children's choices were consistent with an underweighting of low-probability events and an overweighting of high-probability events; choices of adults were more consistent with an objective use of probabilities when evaluating risky prospects. A study by Levin, Weller, Pederson, and Harshman (2007), in which children aged 5–11 and adults chose between gambles differing in EV, also showed age-related increases in EV-maximising choices. In the current study, we extend this developmental picture by assessing the ability of children, adolescents and adults to maximize EV in choosing between pairs of gambles that differ in EV.

Our second goal was to investigate age differences in the impact of risk on choice. Participants’ preference for risk was identified by assessing the contribution of the variance of gambles to behaviour, where a risk-seeking participant is one who shows a tendency to choose gambles with high variance. Because the probabilities and outcomes of gambles were shown to participants, risk-seeking in the present study refers to a true preference for risk. In contrast, seemingly risky behaviour in real life could arise due to a number of factors, including incomplete knowledge of the range of possible outcomes or a reduced ability to judge their probabilities. We predicted an inverted U-shaped pattern of risk-seeking between childhood and adulthood, with its peak in adolescence, similar to that shown in recent studies (Figner et al., 2009; O’Brien & Steinberg, 2009). Figner et al. (2009) showed that adolescents (age 13–19), relative to adults (age 20+), failed to take into account value as well as probability information when making decisions in an emotional context, and this resulted in more risky behaviour. Participants played a game in which cards shown face down were sequentially turned over to try and gain points. Cards could be turned over as long as gains were encountered, but once a loss occurred the trial terminated. A participant must therefore make a decision about when to stop turning over cards, in order to finish with a gain of points. Figner et al. found sub-optimal adolescent decision-making only in an emotional (‘hot’) version of the task, and not in an unemotional (‘cold’) version. Another study showed that, in a driving game played alone or in the presence of peers, adolescents made poor decisions relative to adults, but only when friends were present (O’Brien & Steinberg, 2009; Steinberg, 2008). The friends-present condition can be considered a ‘hot’ version of the driving task, the task used here can also be considered a ‘hot’ task, since it involves making gamble decisions in an emotional context.

The third goal of the current study was to investigate age differences in counterfactually mediated emotions (relief and regret). During the gambling task, participants indicated their emotional response (“How do you *feel* about what just happened?”) to the outcome of each gamble, using a linear rating scale. On half of the trials (complete feedback trials), the outcome for the unselected choice was revealed alongside the outcome for the selected choice, whereas on the other half of trials (partial feedback trials) the outcome of the selected choice only was revealed. Previous work with adults has suggested that the complete feedback condition, with outcome of the unselected choice always revealed, gives rise to a counterfactual comparison between the actual outcome and the outcome of a foregone alternative (what might have been), and that this comparison modulates self-reported emotional responses (Camille et al., 2004; Coricelli et al., 2005). For example, making a choice that yields a win of $100 gives rise to a more positive feeling if the unchosen outcome is revealed as $10 rather than $1000.

The emotions participants experience are known as ‘reward-based’ emotions (Rolls, 2005). These are emotions that are produced by a reinforcer, and depend on the reward contingency (delivery or omission) and on the type of reinforcer. In previous studies using the present task, galvanic skin recordings show that gamble outcomes are accompanied by emotional responses (Camille et al., 2004). We label these responses in a context-dependent manner, according to the counterfactual and reward context in which they occur. Thus, a participant's self-reported emotion in response to a win of points when the unchosen alternative is revealed as a loss of points is labelled as relief. The emotion in response to a loss of points when the unchosen alternative is revealed as a win of points, is labelled as regret. Based on suggestions that the ability to reason counterfactually about the outcomes of decisions continues to develop during childhood and adolescence, we hypothesised age group differences in emotional responses to chosen outcomes as a function of the outcome of the unchosen alternative in complete feedback trials.

## 1 Method

### 1.1 Participants

Pre-adolescent males (aged 9–11; *n* = 20) were from an urban primary school. Adolescent males (aged 12–18; *n* = 52) were from a secondary school in the same area and at which a proportion of school leavers from the primary school enrol each year. The adolescent group was subdivided into young adolescents (aged 12–15.5; *n* = 26) and mid-adolescents (aged 15.5–18; *n* = 26). Adult males (aged 25–35; *n* = 17) were recruited from a database of former students of the secondary school. Therefore, child, adolescent and adult groups were well matched for educational background and socioeconomic status. Participants were restricted to males to reduce variance attributable to sex differences in traits such as sensation-seeking, which might influence risk preferences (Steinberg et al., 2008), and sex differences in brain maturation (Giedd et al., 1999; Neufang et al., 2009). Previous studies have shown higher risk-seeking in males than in females (Van Leijenhorst et al., 2008).

### 1.2 Procedure

**Fig. 1:**
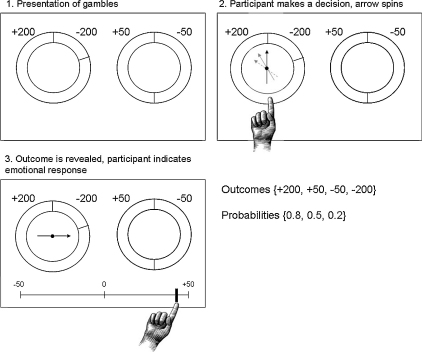
The gambling task: *partial feedback* condition. On each trial, the participant is presented with two gambles (screen 1). The participant chooses one (screen 2). Once the participant has made a choice, the arrow spins round on the chosen gamble until it comes to rest on the obtained outcome (screen 3). Finally, the participant indicates his emotional response to the outcome on a continuous scale from −50 (I feel very negative) to +50 (I feel very positive). In the *complete feedback* condition, the arrows spin round on both wheels although the participant only receives the amount indicated on the chosen wheel.

On each trial, the participant was presented with two ‘wheels of fortune’ on a computer screen (Fig. 1). The participant was instructed to choose one of the wheels, with the aim of maximising the number of points won. Possible wins and losses on each wheel were indicated by positive or negative numbers next to the wheels. Their associated probabilities were indicated by the relative size of the sectors of the wheel. When the participant pressed a button indicating his choice, an arrow spun round on the wheel and came to rest showing the number of points won or lost on that trial. The participant was then asked to indicate how he felt using a linear rating scale at the bottom of the screen: from −50 (extremely negative) to +50 (extremely positive).

The game was played under two conditions, lasting approximately 10 min each, preceded by a 5-trial practice phase. Each condition consisted of 30 self-paced trials, separated by an inter-trial interval of 1 s. In the *partial feedback* condition which occurs first, the participant was shown the outcome of the chosen wheel, but not the outcome of the unchosen wheel. In the subsequent *complete feedback* condition, the participant was shown the outcomes of both wheels, but only gained the number of points indicated by the arrow on the chosen wheel. The task was conducted using Cogent software running in Matlab, which logged behavioural responses.

Possible outcomes took discrete values of +200, +50, −50 or −200 points. Outcome probabilities for each wheel were 0.2/0.8 or 0.5/0.5. The two alternatives always differed in EV and in the value of their actual outcomes. Otherwise, pairs of gambles and the combination of probabilities and outcomes for each gamble were split approximately evenly among possible combinations. Thus, the EV of all 120 gambles ranged from −170 to +170, with a mean and median close to or at zero (mean EV = −8, median EV = 0), and a range of differences in EV between the paired gambles across the 30 trials in each condition from −195 to +195 (pairings of extreme good EV gambles with extreme bad EV gambles were avoided as this sort of trial is experienced as an ‘obvious’ choice), with a mean and median close to zero (mean *d*EV = 3, median *d*EV = 12). The set of pairs of gambles and the order in which they were presented was the same in each condition.

The task was administered individually to child and adolescent participants in a quiet room at school, and at the home or workplace of adult participants. Duration was 15–25 min, depending on participant reaction times.

### 1.3 Data analysis

Datapoints lying >3 s.d. from the grand mean of the dependent variable in each analysis were considered outliers and were excluded from that analysis.

#### 1.3.1 Logit regression analysis: the effect of expected value and risk on choice

Decision-making was analysed by regression analysis with a panel logit procedure (Camille et al., 2004; Coricelli et al., 2005). Both random and conditional fixed effects were estimated, and we report the results for the random effects analysis. This procedure treats each participant as a unit and each subsequent trial as time and thus the dataset as a sequence of related observations from several units (participants) identified by the panel variable. The analysis then identifies the maximum likelihood of a model of the data that takes into account decision variables, in this case the difference in EV and risk (variance) between gambles.

In our model, *x*_1_ and *y*_1_ represent the highest and the lowest outcome of gamble 1 (*g*_1_, the left-hand wheel), and *x*_2_ and *y*_2_ represent the highest and the lowest outcome of gamble 2 (*g*_2_, the right-hand wheel). The probability of *x*_1_ is *p* and the probability of *y*_1_ is 1 − *p*; the probability of *x*_2_ is *q* and the probability of *y*_2_ is 1 − *q*. The probability of choosing gamble 1 is estimated as:

(1) $$\mathrm{Pr}(g_{1it})=1-\mathrm{Pr}(g_{2it})=F[d\text{E}{\text{V}}_{it},d\text{S}{\text{D}}_{it}]$$

where *i* = individual, *t* = time and the function *F*[*θ*] denotes the function *e*^*θ*^/(1 + *e*^*θ*^). We investigated the effect on choice of the difference in EV and risk between paired gambles ordered in time, defining the variables *d*EV and *d*SD as:

(2) $$d\text{EV}=\text{EV}(g_{1})-\text{EV}(g_{2})=[(px_{1}+(1-p)y_{1})-(qx_{2}+(1-q)y_{2})]$$

(3) $$d\text{SD}=\text{stdev}(g_{1})-\text{stdev}(g_{2})$$

where:

(4) $$\text{stdev}(g_{1})=\surd [p{\left(x_{1}-\text{EV}(g_{1})\right)}^{2}+q{\left(y_{1}-\text{EV}(g_{1})\right)}^{2}]$$

A participant who maximizes EV and risk will choose *g*_1_ if [EV(*g*_1_) > EV(*g*_2_)] (Eq. (2)), and if [stdev(*g*_1_) > stdev(*g*_2_)] (Eq. (3)).

To ascertain whether the impacts of EV and risk on behaviour were dependent on age, we first orthogonalised the two variables *d*EV and *d*SD (attributing all the common variance to *d*EV^1^) and then modelled interactions between age and each of the orthogonalised parameters *d*EV and *d*SD. We tested the hypothesis that risk-seeking would show an inverted U-shaped pattern across age by modelling the interaction between a quadratic function of age (age^2^) and the impact of risk (*d*SD) on behaviour; we also tested for a quadratic relationship between age and EV. In order to directly compare the models testing for linear and quadratic effects of age on the variables *d*EV and *d*SD, we conducted a likelihood-ratio test.

The outputs of the logit regressions show the coefficients of model parameters (*d*EV, *d*SD) and their associated significance levels. A coefficient that is not significantly different from zero indicates that participant behaviour is neutral with respect to that coefficient. For example, participants could be risk-neutral, or insensitive to EV. A coefficient that is significantly different from zero indicates that participants tend to maximise or minimise the decision variable. The sign of the coefficient indicates whether the decision variable increases (+) or decreases (−) the probability of choice. For example, a high, positive coefficient for risk (*d*SD) indicates that a more risky choice (one with higher outcome variance) has a higher probability of being chosen. We tested for significance of the variables *d*EV and *d*SD across participants and within each age group separately, setting the threshold for significance at *p* < 0.05.

We first report a simple model, in which the utility function is estimated using the decision variables *d*EV and *d*SD (Table 1a). We subsequently add the age variable and its interaction with *d*EV and *d*SD, before finally testing for quadratic effects by including interactions between age^2^ and the variables *d*EV and *d*SD. We tested whether the addition of each variable improved the model and report a reduced model including only the variables that significantly explain choice (Table 1b).

#### 1.3.2 Proportion of EV and risk-maximising choices by age group

To complement results from the logit regression analyses, which treat age as a continuous variable and therefore may be influenced by the distribution of age, we calculated the proportion of trials for which each of the four age groups selected the gamble that maximized EV or risk. One-way analysis of variance (ANOVA) was used in each case to test for age group differences, with a *p* < 0.05 threshold for significance and Bonferroni corrections applied to *post hoc* tests. We also interrogated the curve resulting from a regression between age^2^ and the proportion of risky choices, to locate the point of inflection, which is the age at which the proportion of risky choices is maximal.

#### 1.3.3 Other indices of performance

Mean winnings closely follow the proportion of EV-maximising choices, since a participant who maximizes EV will win more points. However, we additionally investigated the relationship between participant age and mean winnings by conducting linear regression analysis. We performed two regressions, one on the entire group (children, adolescents and adults) and a second on only children and adolescents, to rule out the possibility that this effect was driven by two discrete clusters in age and because we hypothesised that the major differences in performance would occur in this age range.

To ascertain whether any effects of age on performance were due simply to slower learning of the task at younger ages (Crone & van der Molen, 2007), rather than to differences in strategy, we conducted linear regression analyses between age and the difference in mean winnings between the first and second 15 of the 30 trials per condition, as well as one-way ANOVA on the mean difference in winnings between the first and second halves of each condition, with age group as a between-subjects factor.

#### 1.3.4 Strength of counterfactually mediated emotions and overall emotional response by age group

We hypothesised that the effect on emotion ratings of a counterfactual comparison between the chosen and the unchosen outcome would differ by age. To test this hypothesis, we selected trials for which a counterfactual comparison could either enhance or diminish the degree of satisfaction a participant would feel. That is, we selected trials from the complete feedback condition for which the obtained outcome was either +50 or −50, and the unchosen alternative was either +200 or −200. To clarify, consider a counterexample: if a participant wins +200 points, any unobtained outcome (+50, −50 or −200) would have been worse; the counterfactual comparison will always be in the downward direction and will always be confounded with outcome magnitude. In contrast, a win of −50 appears satisfactory if the unobtained outcome is revealed to be −200, but unsatisfactory if the unobtained outcome is revealed to be +200. Emotion ratings from the latter trial types were therefore used to examine age group differences, using one-way ANOVA with a threshold for significance at *p* < 0.05, and Bonferroni corrections to *post hoc* *t-*tests.

We investigated overall differences in emotion reports by age and condition by conducting mixed model repeated-measures 2 × 2 × 4 ANOVA on emotion reports, with Bonferroni-corrected *post hoc* *t-*tests to investigate simple effects. Because order effects (the partial condition always occurred first) may contribute to a main effect of feedback or interactions thereof, we report only those age-independent effects that replicate previous results (Bault, Coricelli, & Rustichini, 2008; Camille et al., 2004; Coricelli et al., 2005).

## 2 Results

### 2.1 Performance

#### 2.1.1 Behavioural sensitivity to the difference in EV between gambles

Logit regression analysis showed that in both conditions, across participants as well as within each age group, the difference in EV between gambles affected choice. Participants exhibited a behavioural preference for gambles with higher EV. Because behaviour with regard to EV was similar across conditions, and in order to increase power, we collapsed across conditions. Thus, for all 89 participants, as well as within each age group, EV coefficient *d*EV > 0, with *p* < 0.001 (Table 1a). However, participant age modulated the extent to which the difference in EV between gambles affected choice (interaction between age and *d*EV coefficient, *d*EV × age > 0, *p* < 0.001; Table 1b), such that the impact of the difference in EV between gambles on choice behaviour increased with increasing age. That is, older participants exhibited a stronger behavioural preference for gambles with higher EV than did younger participants. The interaction between age^2^ and *d*EV was not significant and was therefore removed from the model.

To assess whether the relationship between age and the impact of EV on choice was better described as linear or quadratic, we conducted a likelihood-ratio test. This showed that adding a quadratic component for the interaction between age and *d*EV (*d*EV × age^2^) did not significantly improve the model (likelihood-ratio test: LR *χ*^2^(1) = 1.47, *p* = 0.226). Therefore, we can conclude the effect of age on *d*EV is linear.

#### 2.1.2 Proportion of EV-maximising choices by age group

One-way ANOVA on the proportion of trials for which participants in each of the four age groups chose the gamble with higher EV was not significant (*F*_3,85_ = 2.354, *p* = 0.078).

#### 2.1.3 Relation between age and mean winnings

**Fig. 2:**
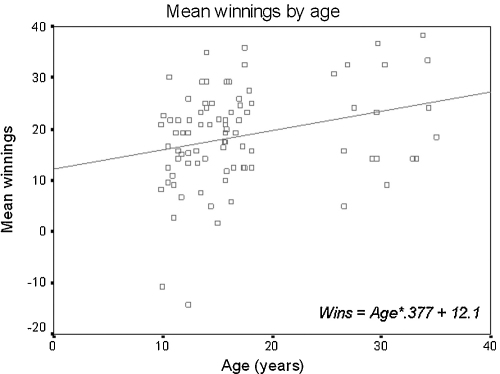
Mean winnings across the task show a positive correlation with age (*β* = 0.280, *r*^2^ = 0.078, *p* = 0.009). Two outliers >3 s.d. away from the mean were excluded. Note that the two lower data points shown in this figure are not outliers by this definition; the linear relationship remains significant when these lower data points are excluded (see text for details).

Linear regression analysis showed that mean winnings across the task were positively correlated with age (mean winnings: *β* = 0.280, *r*^2^ = 0.078, *p* = 0.009; two outliers were excluded: one YA, one MA^2^; see Fig. 2). This result is consistent with the logit regression result showing an age-related increase in EV-maximising behaviour. To rule out the possibility that this effect was driven by two discrete clusters in age, we carried out a second linear regression analysis on mean winnings against age for the child and adolescent groups only. It showed that mean winnings across the task were also positively correlated with age across this age range (*β* = 0.340, *r*^*2*^ = 0.115, *p* = 0.004).

#### 2.1.4 Effect of age on learning

Linear regression between age and the difference in mean winnings between the first and second 15 of the 30 trials per condition, as well as one-way ANOVA on the mean difference in winnings between first and second halves of both conditions, with age group as a between-subjects factor, revealed no significant effect of age (regression *p* = 0.879; no outliers) or age group (*F*_3,84_ = 0.204, *p* = 0.894; one YA excluded due to incomplete partial condition) on the difference in mean winnings between the first and second halves of each condition.

### 2.2 Risk-seeking

#### 2.2.1 Behavioural sensitivity to the difference in risk between gambles

Logit regression analysis showed that across age groups, the difference in risk between gambles (see Eq. (3)) affected choice. Overall, participants exhibited a behavioural preference for more risky gambles, that is, gambles with higher outcome variance (risk coefficient *d*SD > 0, *p* < 0.001; Table 1a). The difference in risk between gambles also predicted choice in each of the three younger groups (risk coefficient *d*SD > 0, *p* = 0.004 for child group, and *p* < 0.001 for YA and MA groups), but not in the Adult group alone (*p* > 0.7). Thus, children and adolescents showed a preference for more risky gambles and adults did not.

#### 2.2.2 Non-linear relation between age and the impact of risk on behaviour

A significant quadratic interaction between age and *d*SD indicated that age influenced the extent to which the difference in risk between gambles affected choice (coefficient *d*SD × age^2^ > 0, *p* = 0.022; Table 1b). Choice was not predicted by a linear function of risk × age (coefficient *d*SD × age: 1, *p* = 0.064; Table 1b). To directly compare linear vs. quadratic effects of *d*SD, we conducted a likelihood-ratio test. It showed that adding a quadratic component for the interaction between age and the risk coefficient (*d*SD × age^2^) to a model only containing the linear component (*d*SD × age) significantly improved the model (likelihood-ratio test: LR *χ*^2^(1) = 5.31, *p* = 0.021). Therefore, we can conclude the effect of age on the propensity to seek risk is quadratic.

#### 2.2.3 Proportion of risk-maximising choices by age group

**Fig. 3:**
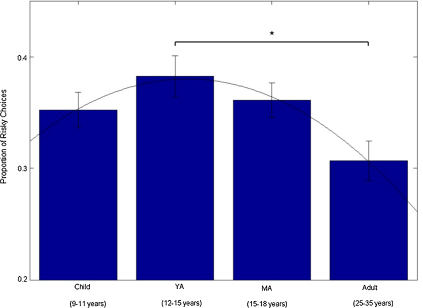
The proportion of risky choices differed across age groups (one-way ANOVA: *F*3,85 = 3.077, *p* = .032). The young adolescent group made a significantly greater proportion of risk-maximising choices than did the adult group (paired comparisons: *p* = .021 for YA vs. adult). The graph is fit with a quadratic function for purposes of illustration; a quadratic fit between age as a continuous variable and proportion of risky choices showed a point of inflection at age 14.38 years, and logit regression revealed an inverted U-shape relation between age and the influence of risk on choice (Table 1b).

One-way ANOVA on the proportion of trials for which participants in each age group chose the gamble with higher risk was significant, *F*_3,85_ = 3.077, *p* = 0.032. Fig. 3 shows the proportion of risky choices by age group. For the purposes of illustration, a quadratic function has been fit to the group data. Paired *post hoc* Bonferroni corrected comparisons showed that the YA group made a significantly greater proportion of risk-maximising choices than did the adult group (*p* = 0.021; all other comparisons *p* > 0.2). Interrogation of the curve resulting from a regression between age^2^ as a continuous variable and the proportion of risky choices revealed that the point of inflection was located at 14.38 years. That is, 14.38 is the age at which participants made the greatest proportion of risky choices.

### 2.3 Emotion ratings following outcomes

#### 2.3.1 Strength of counterfactually mediated emotion by age group

**Fig. 4:**
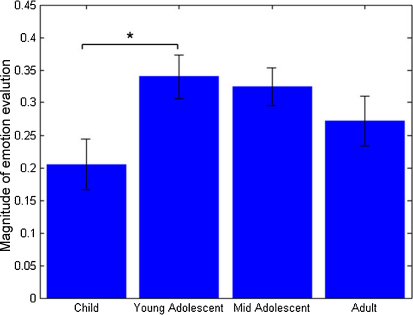
The strength of counterfactually mediated emotion ratings (relief and regret) is greater in the young adolescent than in the child group as shown by one-way ANOVA (*F*3,83 = 3.155, *p* = 0.029) with Bonferroni-corrected paired post hoc tests (child vs. young adolescent mean difference = 6.74, *p* = 0.037).

One-way ANOVA showed a significant effect of age group (*F*_3,83_ = 3.155, *p* = 0.029; two outliers were excluded^3^) on the strength (magnitude) of counterfactually mediated reported emotions (relief and regret). We considered counterfactually mediated emotions to be the emotion ratings elicited in response to a win of ±50 when the unobtained outcome was −200 (relief) or +200 (regret). Paired *post hoc* Bonferroni-corrected comparisons revealed significantly weaker counterfactually mediated emotion ratings in the child than in the YA group (mean difference 6.74, *p* = 0.037), while no other comparisons reached significance (Fig. 4).

In contrast, investigation of overall differences in emotion intensity across age and condition showed no evidence of stronger emotion ratings in adolescence, nor any interactions between age and within-groups factors. This suggests that the age group difference in emotion ratings reported above is specific to reported emotions elicited in response to a counterfactual comparison between chosen and unchosen alternatives. We did, however, replicate previous findings (Bault et al., 2008; Coricelli et al., 2005) by showing an influence on emotion ratings of valence (*F*_1,83_ = 400.45, *p* < 0.001) and feedback (*F*_1,83_ = 9.84, *p* = 0.002), and an interaction between these factors (*F*_1,83_ = 39.82, *p* < 0.001).

#### 2.3.2 *Post hoc* tests on emotion and risk

We conducted *post hoc* *t*-tests on child vs. YA differences in counterfactually mediated emotion ratings separately for positive and negative outcomes to investigate whether the stronger emotion ratings in YA relative to children were driven by a hyper-responsiveness to positive rather than negative outcomes (Casey et al., 2008; Ernst et al., 2005; Eshel et al., 2007). Independent samples *t*-tests revealed higher YA than child emotion ratings on relief (‘lucky escape’) trials (*t*_43_ = −2.22, *p* = 0.02, 1-tailed), whereas the same comparison for regret trials was not significant (*t*_43_ = .73, *p* = 0.24, 1-tailed). This *post hoc* result suggests that the YA group showed an enhanced emotional response to relief or ‘lucky escape’ outcomes relative to the child group.

To assess whether this difference predicted risk-taking, we conducted linear regression analyses between the emotion response on relief trials and the proportion of risky choices. This analysis was not significant for the full sample (*β* = 0.176, *r*^2^ = 0.031, *p* = 0.103), but was significant within child and YA groups together (*β* = 0.305, *r*^2^ = 0.093, *p* = 0.041), and was marginally significant within child, YA and MA groups together (*β* = 0.228, *r*^2^ = 0.052, *p* = 0.056). It was not significant in the adult group alone (*β* = 0.067, *r*^2^ = 0.005, *p* = 0.804). This suggests that children and adolescents become less cautious in their choices after surprisingly lucky (relieving) wins. However, this conclusion is tentative and requires further investigation because these correlations do not survive Bonferroni correction for multiple comparisons.

## 3 Discussion

Our results showed that among males in a probabilistic gambling task, the ability to maximise EV increased linearly with age, whereas risk-seeking showed a quadratic relationship with age and the proportion of risky choices peaked in mid-adolescence. This is one of the first behavioural studies to provide evidence for an inverted U-shaped relation between age and risky decision-making. In addition, we found an increase between childhood and adolescence in the strength of counterfactually mediated reported emotions, although individual emotion ratings did not predict risky decision-making across age.

### 3.1 Development of optimal decision-making: sensitivity to EV

Participants made a series of decisions between paired gambles that differed in EV, the sum of possible outcomes of a gamble weighted by their probabilities. All age groups showed evidence of taking EV into account when making decisions. At all ages, decisions were predicted by a model in which gambles with high EV were preferentially chosen over gambles with low EV, a result consistent with previous studies (see Boyer, 2006, for review).

However, the extent to which participants maximised EV varied by age (Table 1b). Older participants were more likely to make decisions that maximised EV. Since mean winnings closely follow the proportion of EV-maximising choices, a positive correlation between age and mean winnings was found. Therefore, performance on the task improved with age (Fig. 2). As shown by analysis of the difference in winnings between the first and second 15 of the 30 trials per condition, this age trend was not due simply to slower learning of the task at younger ages, but is likely to reflect stable age differences in strategy.

Our results are in agreement with findings from previous studies. In a behavioural economic study conducted by Harbaugh et al. (2002) in which participants aged 5–64 years chose between a certain outcome and a gamble with equivalent EV, the proportion of optimal, unbiased choices increased with age throughout childhood and adolescence. A study by Levin et al. (2007) showed similar age-related increases in the tendency to maximise EV among children (aged 5–7), pre-adolescents (aged 8–11) and adults. The present study extends this developmental picture by showing that the ability to maximize EV when choosing between two gambles (rather than a gamble and a sure thing) shows linear development during late childhood and adolescence.

A recent study conducted by Figner et al. (2009) showed that the propensity to take into account relevant probability and reward information continued to mature between adolescence (age 13–18) and adulthood (20+). Interestingly, this developmental difference was only found using an emotional (‘hot’) version of the task. In an unemotional (‘cold’) version of the same task, there were no age differences. Due to the emotional component of our gambling task (participants reported their emotional response to gamble outcomes, including unchosen ones), it is may be similar to the ‘hot’ task of Figner et al. (2009) and is therefore in agreement with a previous study. However Van Leijenhorst et al. (2008) found no age differences between 8 and 30 years in the ability to maximise reward in a wheel of fortune task. Further studies are needed to clarify the source of this discrepancy, but a suggestion could be the difference in extent to which the attention of participants was directed to unchosen or unobtained outcomes. Development in the ability to maximise EV found here may be partially due to the amplified emotional responses elicited by the comparison between the actual outcome and ‘what might have been’.

### 3.2 Developmental profile of risk-taking

In the present task, paired gambles differed in risk (measured as outcome variance; Bossaerts & Hsu, 2008). The child group and both adolescent groups showed evidence of taking risk into account when making decisions. In these age groups, decisions were predicted by a model in which gambles with high risk were preferentially chosen over gambles with low risk. In contrast, the adult group showed no evidence of taking risk into account when making decisions; adults were risk-neutral.

The extent of risk maximisation differed by age. There was an inverted U-shaped relationship between age and the influence of risk on decision-making. Logit regression showed that the impact of the difference in risk between alternatives increased with age at the younger end of the age range. Toward the middle of the age range, the increase in impact of the difference in risk started to decrease with age. By adulthood, the effect was reversed. That is, adolescents showed the strongest tendency to select high-risk gambles. In addition, there was a significant effect of age on the proportion of trials for which participants selected the high risk alternative. Young adolescents (aged 12–15) made a significantly greater proportion of risky choices than did adults, and the age at which risky choices peaked was 14.38 years (Fig. 3).

These results expand on those from two previous studies that have shown that the tendency to make risky decisions in emotional gambling tasks decreases between adolescence and adulthood (age 11–15 vs. age 21–31, Eshel et al., 2007; age 13–19 vs. age 20+, Figner et al., 2009). However, it has been suggested that the tendency to make risky decisions shows an inverted U-shaped relation to age, from childhood through adolescence and into adulthood. Ours is one of the first empirical studies to demonstrate such an inverted U-shaped relationship, using a behavioural task assessing decision-making under risk. This result complements theoretical and observational studies. Epidemiological studies show a peak during adolescence in ‘risky’ activities such as dangerous driving (Eaton et al., 2008), which has led to the suggestion of an inverted U-shaped relationship between age and the propensity to be risk-seeking, with its peak in adolescence (Casey et al., 2008; Steinberg et al., 2008). However, choosing to engage in real life ‘risky’ activities is likely to have a variety of underlying causes and as such does not constitute a preference for risk in a behavioural economic sense. The present study is an important step toward clarifying the conditions under which adolescents take risks relative to children as well as adults, as it has shown an experimental context in which adolescents appear risk-seeking.

### 3.3 Development of the counterfactually mediated emotions of relief and regret

In this study, participants rated their emotional response to the outcome of each gamble. On half of the trials (*complete feedback* condition) they were shown the outcome for the unchosen, as well as chosen, wheel. Previous work with adults has shown that such feedback gives rise to a counterfactual comparison between the outcome of a choice (reality) and its foregone alternative (what might have been), and that this comparison influences participants’ emotional responses as well as their subsequent behaviour (Coricelli et al., 2005). We investigated age differences in the emotional response to situations in which the outcome for the unchosen gamble was revealed as either better (resulting in regret) or worse (resulting in relief) than that for the chosen gamble. The magnitude of relief and regret differed by age, with young adolescents (aged 12–15) reporting stronger emotions than children (aged 9–11). This result is consistent with the suggestion that the ability to reason counterfactually about the outcomes of decisions is still developing during adolescence (Baird & Fugelsang, 2004).

*Post hoc* tests indicated that the increased magnitude of counterfactually mediated emotions in young adolescents relative to children was driven by a more strongly positive response to relief outcomes, which can be conceptualised as ‘lucky escapes’. This result is compatible with a developmental picture of heightened reward sensitivity in adolescents relative to children (Steinberg et al., 2008). Further studies are needed to replicate and extend this developmental finding. A preliminary suggestion is that adolescents are prone to make incautious decisions after surprisingly lucky (relieving) outcomes.

### 3.4 Relations between emotions and risk-taking

Our findings lend weight to the suggestion that risk-taking in adolescence cannot solely be explained by an inability to gauge the most advantageous course of action, for if this were the case children would take more risks. As has been suggested elsewhere (Casey et al., 2008; Steinberg, 2008), adolescent risk-taking arises due to an interaction between two separate developmental trends—a gradual improvement in cognitive control-related abilities (here contributing to improvements in the ability to maximize EV), superimposed upon non-linear trajectories of sensation-seeking and socio-emotional factors. We found that the increase in risk-taking coincided with an increased strength of counterfactually mediated emotions (in particular, relief). However, individual emotion scores did not strongly predict risk-taking. This may reflect a lack of power in the present study; alternatively, some other measure of emotional responding, or a cognitive variable (Galvan, Hare, Voss, Glover, & Casey, 2007), may be a more proximal predictor of individual risk-taking. Future studies designed to test the relationship between risk-taking and the emotional response to a ‘lucky escape’ will shed more light on this issue.

Distinct developmental trajectories for EV-maximising vs. risk-taking suggest that adolescents do not engage in risky behaviours simply because they are unable to estimate the negative consequences of their decisions. We found that young adolescents showed an enhancement of the emotional response to relief outcomes (a ‘lucky escape’) relative to children, although they did not differ from adults on this measure. We presented tentative evidence that these relief responses predict risky choices. However, further studies are needed to establish which social-emotional and cognitive factors most strongly contribute to adolescent risk-taking both in females and in the males studied here.

## Figures and Tables

**Table 1:** Participant choice behaviour as a function of the difference in expected value (*d*EV) between gambles and the difference in risk (*d*SD) between gambles (a), and as a function of these and other variables (b).

Variable name	Coefficient	Standard error	*Z*	*P*	[95% conf. interval]	
(a)						
Difference in expected value: *d*EV	2.1903	0.0580	37.74	<0.001	2.0765	2.3040
Difference in risk: *d*SD	0.3279	0.0325	10.09	<0.001	0.2642	0.3916
Constant	0.2272	0.0383	5.94	<0.001	0.1522	0.3022
Log likelihood = −2151.0887, Wald *χ*^2^ = 1453.61, Prob > *χ*^2^ = 0.000						

(b)						
Difference in expected value: *d*EV	1.3480	0.1711	7.88	<0.001	1.0126	1.6833
Difference in risk: *d*SD	−0.1295	0.3178	−0.41	0.684	−0.7523	0.4933
Interaction between EV and age: *d*EV × age	0.0522	0.0105	5	<0.001	0.0318	0.0727
Interaction between risk and age: *d*SD × age	0.0620	0.0335	1.85	0.064	−0.0037	0.1278
Interaction between risk and age^2^: *d*SD × age^2^	−0.0018	0.0008	−2.3	0.022	−0.0033	−0.0003
Constant	0.2292	0.0385	5.95	<0.001	0.1537	0.3047
Log likelihood = −2127.5693, Wald *χ*^2^(3) = 1413.97, Prob > *χ*^2^ = 0.000						

*Note*. *d*EV and *d*SD are orthogonalised and the common variance attributed to *d*EV. Data are from all trials, totalling 5289 observations and 89 participants.

## Footnotes

1 Reversing the direction of orthogonalisation led to similar results.

2 Note that this result is the same when the two outliers’ data are included (*β* = 0.261, *r*^2^ = 0.068, *p* = 0.001), and the relationship remains significant when the two lower data points shown in Fig. 2 (which lie within 3 s.d. of the mean and are thus not outliers by our definition) are excluded (*β* = 0.265, *r*^2^ = 0.070, *p* = 0.015).

3 Outliers were one Child whose data lay +3.21 s.d. away from the mean, and one Adult whose data lay +4.31 away from the mean.
